# Decellularized human periodontal ligament for periodontium regeneration

**DOI:** 10.1371/journal.pone.0221236

**Published:** 2019-08-15

**Authors:** Hyoju Son, Mijeong Jeon, Hyung-Jun Choi, Hyo-Seol Lee, Ik-Hwan Kim, Chung-Min Kang, Je Seon Song

**Affiliations:** 1 Department of Pediatric Dentistry, College of Dentistry, Yonsei University, Seoul, Republic of Korea; 2 Oral Science Research Center, College of Dentistry, Yonsei University, Seoul, Republic of Korea; 3 Department of Pediatric Dentistry, College of Dentistry, Kyunghee University, Seoul, Republic of Korea; Università degli Studi della Campania, ITALY

## Abstract

Regenerating the periodontal ligament (PDL) is a crucial factor for periodontal tissue regeneration in the presence of traumatized and periodontally damaged teeth. Various methods have been applied for periodontal regeneration, including tissue substitutes, bioactive materials, and synthetic scaffolds. However, all of these treatments have had limited success in structural and functional periodontal tissue regeneration. To achieve the goal of complete periodontal regeneration, many studies have evaluated the effectiveness of decellularized scaffolds fabricated via tissue engineering. The aim of this study was to fabricate a decellularized periodontal scaffold of human tooth slices and determine its regeneration potential. We evaluated two different protocols applied to tooth slices obtained from human healthy third molars. The extracellular matrix scaffold decellularized using sodium dodecyl sulfate and Triton X-100, which are effective in removing nuclear components, was demonstrated to preserve an intact structure and composition. Furthermore, the decellularized scaffold could support repopulation of PDL stem cells near the cementum and expressed cementum and periodontal-ligament-related genes. These results show that decellularized PDL scaffolds of human teeth are capable of inducing the proliferation and differentiation of mesenchymal stem cells, thus having regeneration potential for use in future periodontal regenerative tissue engineering.

## Introduction

The regeneration of periodontal tissue is the aim of periodontal therapy to ensure the healing of traumatized teeth. Several kinds of stem cells such as dental pulp stem cells (DPSCs), stem cells from the apical papilla, dental follicle precursor cells, periodontal ligament stem cells (PDLSCs), and stem cells from human exfoliated deciduous teeth (SHED) have been evaluated for their usefulness in the regeneration of dental tissue[1–4]. Periodontal ligament (PDL) tissue contains multipotent stem cells that exhibit a self-renewing capacity to differentiate into various types of cells to form PDL, cementum, and alveolar bone [5, 6]. Stem cells derived from PDL cells have been shown to exhibit superior regenerative properties compared with other cells derived from gingival connective tissue and alveolar bone cells[7].

There have been many attempts to regenerate periodontium, such as using cell- and scaffold-based engineering, but no effective method for regenerating periodontal tissue has yet been reported [8–10]. The various attempts made to achieve PDL regeneration have had limitations, such as gene therapy possibly inducing (human) host responses and tumorigenesis, growth factors being unstable, and biomaterial being likely to fail.

The selection of an appropriate scaffold material is crucial in tissue engineering. The principal functions of the scaffold are to maintain mechanical integrity, supply growth factors, control cell growth, and induce cell differentiation. Commonly used scaffolds such as ceramics, synthetic polymers, and natural polymers have highly adjustable mechanical properties and good production repeatability, but they show low bioactivity [11]. Since extracellular matrix (ECM) scaffolds prepared by the decellularization of mammalian tissues show no immune responses and inherently contain the tissue-specific factors involved in cell growth and differentiation [12, 13], they have been used in studies of regenerative medicine and dentistry [14]. Clinical products derived from the decellularization of tissues are currently used for the replacement and reconstruction of organs and tissues, including human dermis, porcine urinary bladder, human pericardium, and porcine heart valves [15].

Various studies have attempted to apply scaffolds in the field of dentistry. Human dental pulp ECM scaffold that was successfully decellularized promotes the proliferation and differentiation of autologous mesenchymal stem cells [16]. The use of decellularized PDL cell-sheet constructs for periodontal regeneration has also been reported [17, 18]. Decellularized human PDL (dHPDL) cell sheets were shown to maintain the structural integrity of the ECM, retain growth factors, and support allogenic cell repopulation in vitro [17]. Decellularized PDL cell-sheet constructs that promote the differentiation of PDL and mesenchymal stem cells were also shown to support periodontal attachment in a rat periodontal defect model [18]. However, although the application of decellularized PDL tissue to cell sheets has been investigated, the use of human teeth including hard tissue has not been.

A decellularization protocol can be applied to the delayed replantation of an avulsed tooth, autologous transplantation, and periodontal therapy, via the decellularization of PDL tissue following recellularization with PDL stem cells (PDLSCs) derived from orthodontically extracted teeth, primary teeth, and supernumerary teeth. The aim of this study was to apply and evaluate a decellularization protocol that can retain structural integrity and remove cell and DNA effectively, and to test the periodontal recellularization potential of the decellularized human tooth model in vitro.

## Materials and methods

### Tooth-slice sample preparation and cell culture

Third molars that were free of caries and restorations were collected randomly from patients aged 17 to 25 years under approved guidelines set by the Institutional Review Board of the Dental Hospital, Yonsei University (approval no. #IRB 2-2016-0030). Teeth were washed with phosphate-buffered saline (PBS; Invitrogen, Carlsbad, CA, USA) and frozen at –80°C until use. After thawing at room temperature, the teeth were submerged in 0.5% chloramine-T (Sigma-Aldrich, St. Louis, MO, USA) for 2 hours at 4°C, followed by washing in cold running water. The pulp tissues were removed with a barbed broach, and tooth slices were prepared using an precision saw (IsoMet 1000, Buehler, Lake Buff, IL, USA) as described previously (Cordeiro et al. 2008). Samples were collected in cold PBS and immediately subjected to the decellularization procedures.

Previously characterized PDLSCs at passages 3–6 were used in repopulation experiments in a basal cell culture medium comprising alpha minimum essential medium (Invitrogen) containing 10% fetal bovine serum (Invitrogen), 1% L-glutamine/penicillin/streptomycin solution (Invitrogen), and 0.2% amphotericin B solution (Invitrogen) at 37°C in 5% CO_2_.

### Decellularization and repopulation

Based on a review of the literature and a previous study[16], two decellularization protocols were evaluated in this study, as described in Table 1: Protocol I (PI) and Protocol II (PII). All treatment steps were applied to tooth slices at room temperature with constant gentle agitation of the samples in an orbital shaker (SH30, Fine PCR, Gunpo, Gyeonggi, Korea) in the presence of protease inhibitor cocktail (EMD, Millipore, Darmstadt, Germany). At the end of each protocol, samples were rinsed with 10% ethylenediaminetetraacetic acid (EDTA, Fisher Scientific, Houston, TX, USA) at pH 7.4 for 5 min, followed by three rinses of 10 min each with PBS (Invitrogen).

**Table 1:** The tested decellularization protocols.

Protocol	Treatment
Protocol I	2% Triton X-100 and 0.1% NH_4_OH for 72 hours
Protocol II	Three cycles of 1% SDS for 24 hours and 1% Triton X-100 for 24 hours

SDS, sodium dodecyl sulfate.

For repopulation of the decellularized tooth slices (dHPDL), PDL at a density of 1×10^7^ cells/mL in rat tail collagen I (Col I; Corning, Corning, NY, USA) was pipetted directly onto dHPDL using PII or into empty wells (control) in 12-well culture plates (Corning). After 1 hour, 1 ml of basal culture media was applied to each well, and this was changed every 3 days. Cells were cultured at 37°C in 5% CO_2_ for either 2 or 5 weeks.

### Residual DNA quantification

Immediately after each decellularization protocol, samples (n = 10~15) were homogenized and residual DNA was isolated using the QIAamp DNA Investigator Kit (Qiagen, Valencia, CA, USA) and quantified using a spectrophotometer (NanoDrop NE-2000, Thermo Scientific, Waltham, MA, USA).

### Scanning electron microscopy

Samples from the control, PI, and PII groups were incubated in fixative solution (2% paraformaldehyde [PFA], 2% glutaraldehyde, and 0.5% calcium chloride) and then postfixed in 1% osmium tetroxide. Next, the samples were dehydrated sequentially in a graded series of ethanol solutions, coated with platinum, and visualized using a scanning electron microscope (SEM; Hitachi S-3000N, Hitachi Science Systems, Tokyo, Japan) at an accelerating voltage of 20.0 kV.

### Cell viability analysis

The survival of PDL seeded onto dHPDL from PII (n = 10) was compared after 2 weeks of culture. dHPDL without cell seeding served as a negative control. Samples were incubated with the Cell Counting Kit-8 assay (Dojindo Laboratories, Kumamoto, Japan) for 1 hour, and the quantity of water-soluble colored formazan formed by the activity of dehydrogenases in living cells was measured using a spectrophotometer (Benchmark Plus microplate spectrophotometer, Bio-Rad Laboratories, Hercules, CA, USA) at 450 nm. All samples were run in triplicate.

### Histological and immunohistochemistry staining

For histological staining, tooth slices with intact control PDL, dHPDL derived from PI and PII, or repopulated decellularized PDL were fixed with 4% PFA for 1 h, decalcified with EDTA (pH 7.4; Fisher Scientific) for 6 weeks at room temperature, embedded in paraffin, sectioned at a thickness of 4 μm, and stained with hematoxylin and eosin (H&E).

The expression levels of Col I, collagen XII (Col XII), fibronectin, osteocalcin (OC), and cementum protein 23 (CP23) were evaluated by immunohistochemistry (IHC). Protease K (Dako, Carpinteria, CA, USA) was used to retrieve the antigen for the OC and CP23 staining, while no such treatment was performed for Col I, Col XII, and fibronectin. In brief, samples from the control group and dHPDL derived from PI and PII were immersed in 3% hydrogen peroxide for 10 min to inactivate endogenous peroxidase activity, and then incubated with the primary antibody overnight. The primary antibodies were a 1:10000 dilution of antihuman Col I rabbit monoclonal antibody (ab138492, Abcam, Cambridge, UK), a 1:1000 dilution of antihuman Col XII rabbit polyclonal antibody (sc-68862, Santa Cruz Biotechnology, Santa Cruz, CA, USA), a 1:4000 dilution of antihuman fibronectin rabbit polyclonal antibody (F3648, Sigma-Aldrich), a 1:8000 dilution of antihuman OC rabbit polyclonal antibody (#AB10911, Millipore, Temecula, CA, USA), and a 1:1000 dilution of antihuman CP23 goat polyclonal antibody (sc-164031, Santa Cruz Biotechnology). The sections were subsequently incubated for 20 min with horseradish-peroxidase-labeled polymer conjugated with the secondary rabbit antibody in the EnVision+ system kit (Dako) or for 30 min with the Vectastain Elite ABC Kit (PK-6105, Vector Laboratories, Burlingame, CA, USA; goat IgG, diluted 1:200). The color was developed using 3,3'-diaminobenzidine substrate (Dako) and counterstained with Gill’s hematoxylin solution (Merck, Darmstadt, Germany). Negative control sections were stained in the same manner but without the primary antibody reaction procedure.

### Fluorescence staining

PDL specimens were fixed, permeabilized, and blocked following the Image-iT Fix-Perm kit (Life Technologies, Carlsbad, CA, USA) recommendations. NucBlue Fixed Cell ReadyProbes (Life Technologies) were used for staining the nuclei. Immunoreactivity was visualized with a confocal microscope (Zeiss LSM 700) combined with the Zen software (Carl Zeiss, New York, NY, USA).

### Real-time reverse-transcription polymerase chain reaction assay

After 5 weeks of cell seeding and culture, total RNA was extracted from the repopulated tooth slices and from PDL cultured onto cultured dishes (control group) using the RNeasy Mini Kit (Qiagen) (n = 4 for the control group and n = 12 for each experimental group). RNA (100 ng) was reverse transcribed to synthesize cDNA using the Maxime RT premix kit [oligo d(T)_15_ primer; Intron Biotechnology, Seongnam, Gyeonggi, Korea] according to the manufacturer’s instructions. A quantitative real-time polymerase chain reaction (qPCR) assay was performed with SYBR Premix Ex Taq (Takara Bio, Otsu, Japan) and a real-time polymerase chain reaction (PCR) system (ABI 7300, Applied Biosystems, Carlsbad, CA, USA) as per the manufacturer’s instructions. The qPCR conditions were 95°C for 10 sec followed by 40 cycles of 95°C for 5 sec and 60°C for 30 sec, with a final 5-min extension at 72°C. The sequence and size of the primers used are listed in Table 2. The expressions level of each gene was normalized to that of *GAPDH* (the gene encoding glyceraldehyde-3-phosphate dehydrogenase), and the relative expression levels of the studied genes were calculated using the 2^–ΔΔCt^ method[19].

**Table 2:** Primers used for the real-time polymerase chain reaction assay.

Gene	Primer sequence (5’–3’)	Size (bp)
*ALP*	F: GGACCATTCCCACGTCTCAC R: CCTTGTAGCCAGGCCCATTG	137
*Col XII*	F: CGGACAGAGCCTTACGTGCC R: CTGCCCGGGTCCGTGG	180
*CP23*	F: AACACATCGGCTGAGAACCTCAC R: GGATACCCACCTCTGCCTTGAC	142
*OC*	F: CAAAGGTGCAGCCTTTGTGTC R: TCACAGTCCGGATTGAGCTCA	150
*GAPDH*	F: TCCTGCACCACCAACTGCTT R: TGGCAGTGATGGCATGGAC	100

F, forward; R, reverse; *ALP*, alkaline phosphatase; *Col XII*, collagen XII; *CP23*, cementum protein 23; *OC*, osteocalcin; *GAPDH*, glyceraldehyde-3-phosphate dehydrogenase.

### Statistical analysis

All experiments were performed at least in triplicate. Statistical analysis was performed with SPSS (version 23.0, Chicago, IL, USA). The normality of the data was evaluated using the Shapiro-Wilk test, with a significance criterion of p<0.05. The Kruskal-Wallis test followed by the post-hoc Bonferroni test (Bonferroni correction; p<0.017) was used for comparing residual DNA contents, with a significance criterion of p<0.05. The Mann-Whitney U test was performed for cell viability after repopulation and real-time reverse-transcription PCR, also with a significance criterion of p<0.05.

## Results

### Evaluation of different decellularization protocols of human PDL

**Fig 1:**
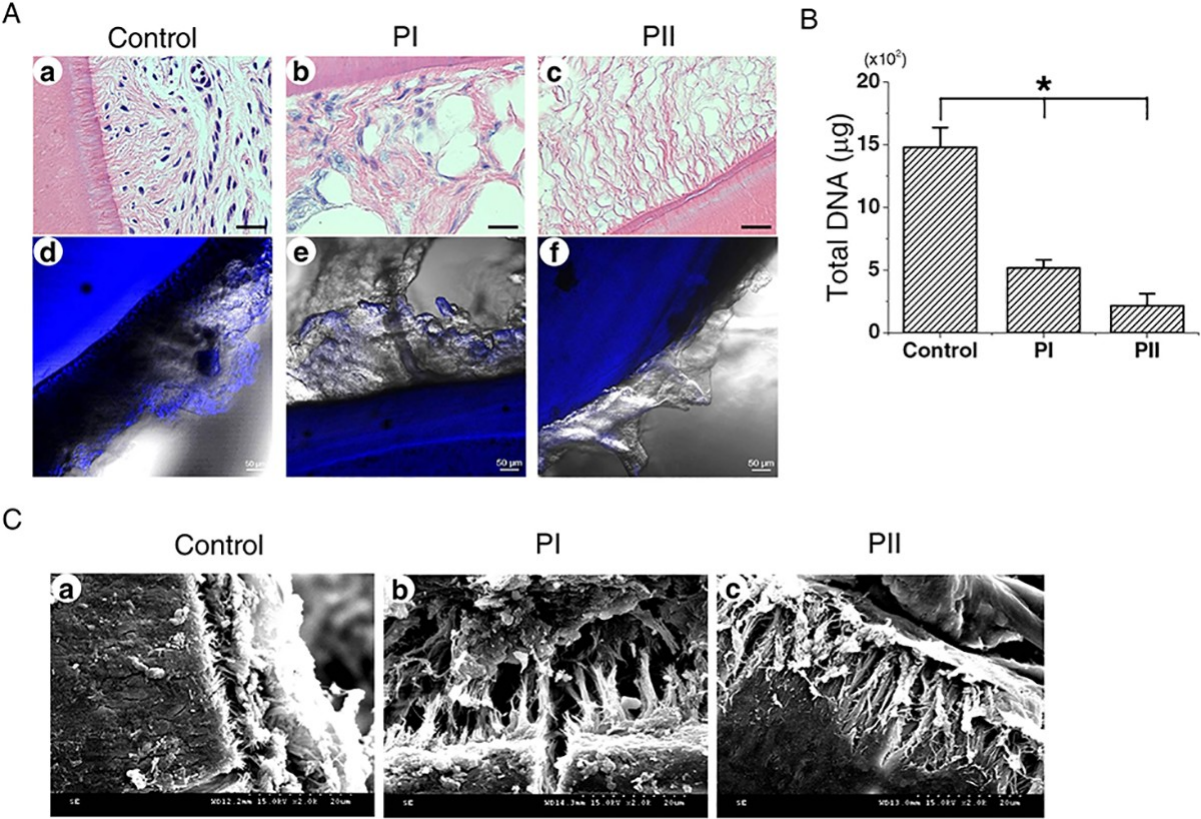
Comparison of different decellularization protocols for periodontal tissue in human tooth slices. (A) Residual nuclei (arrowheads) are indicated by hematoxylin and eosin (H&E) staining (a–c) and fluorescence staining with 4',6-diamidino-2-phenylindole (DAPI) (d–f). More cell nuclei were removed in Protocol II (PII) than Protocol I (PI) after decellularization in H&E and DAPI staining. (B) Residual total DNA contents after decellularization of the periodontal ligament (PDL). The normality of the data was evaluated using the Shapiro-Wilk test (p<0.05). *p<0.05 in Kruskal-Wallis test followed by the post-hoc Bonferroni test (Bonferroni correction; p<0.017). (C) Scanning electron microscopy images of the different protocol groups. Arrowheads indicate remaining Sharpey’s fibers in the PDL. D, dentin. Scale bars: 20 μm in A(a–c), 50 μm in A(d–f), and 20 μm in C.

Residual nuclei and DNA contents after decellularizing PDL were evident compared to the control group not subjected to decellularization (Fig 1A–1C). Compared to the control group, nuclei and DNA were eliminated in the PI and PII groups, and there was a notable DNA reduction for PII of up to approximately 62.32% (Fig 1A and 1B). As seen in H&E and DAPI (4',6-diamidino-2-phenylindole) staining, disperse nuclei were evident for PI (Fig 1A-b and 1A-e), while almost no nuclei were observed for PII (Fig 1A-c and 1A-f). SEM images revealed that some Sharpey’s fibers had been removed, but they had retained their morphology in both the PI and PII groups compared to controls. The density of Sharpey’s fibers was preserved more for PII than for PI (Fig 1C-a and 1C-b).

### ECM characterization of decellularized PDL

**Fig 2:**
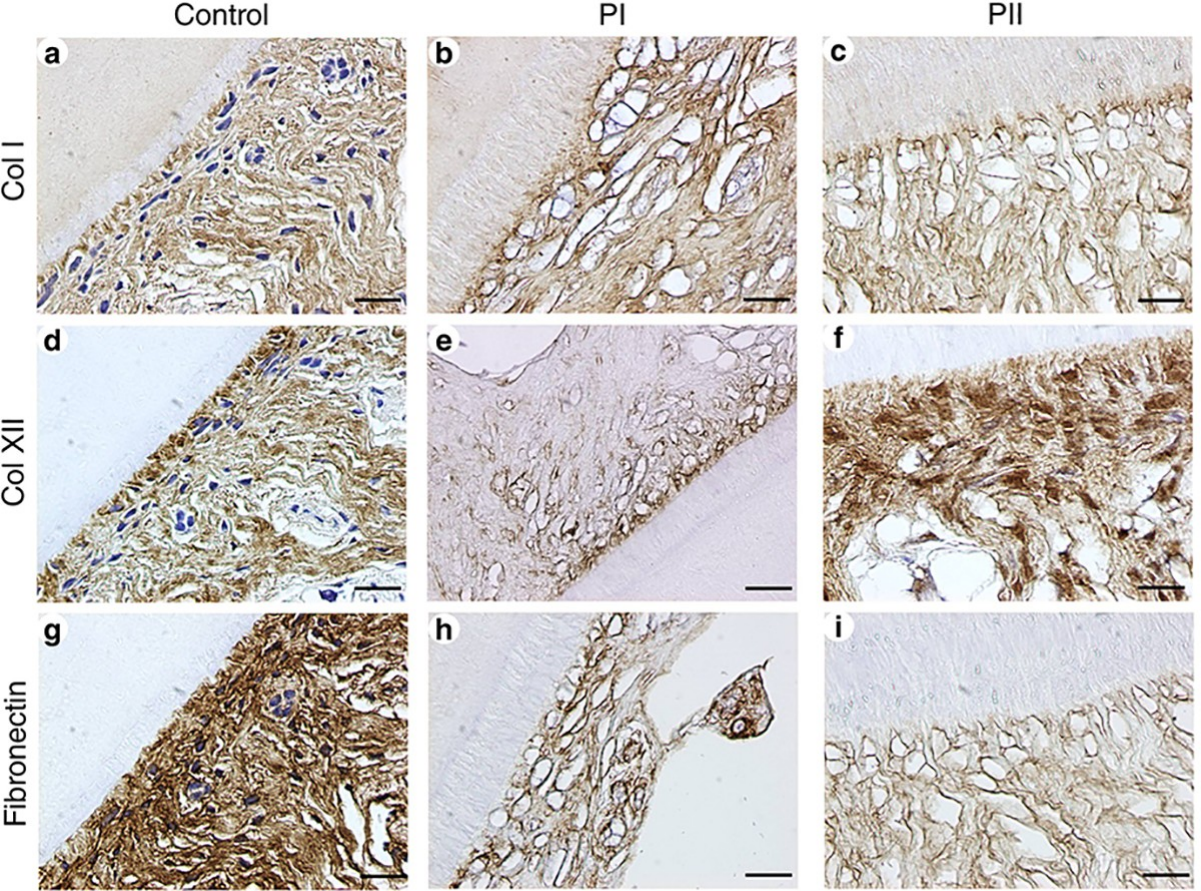
Immunohistochemistry (IHC) staining of collagen I (Col I), collagen XII (Col XII), and fibronectin in the decellularized PDL of tooth slices. Scale bars: 20 μm in a–i.

IHC staining of samples for PI and PII indicated that Col I was almost intact compared with the control group (Fig 2A–2C). Fibronectin was notably diminished for PI and PII (Fig 2G–2I). Col XII was decreased for PI but increased for PII compared to controls (Fig 2D–2F).

### Recellularization potential of human PDLSCs in decellularized PDL

**Fig 3:**
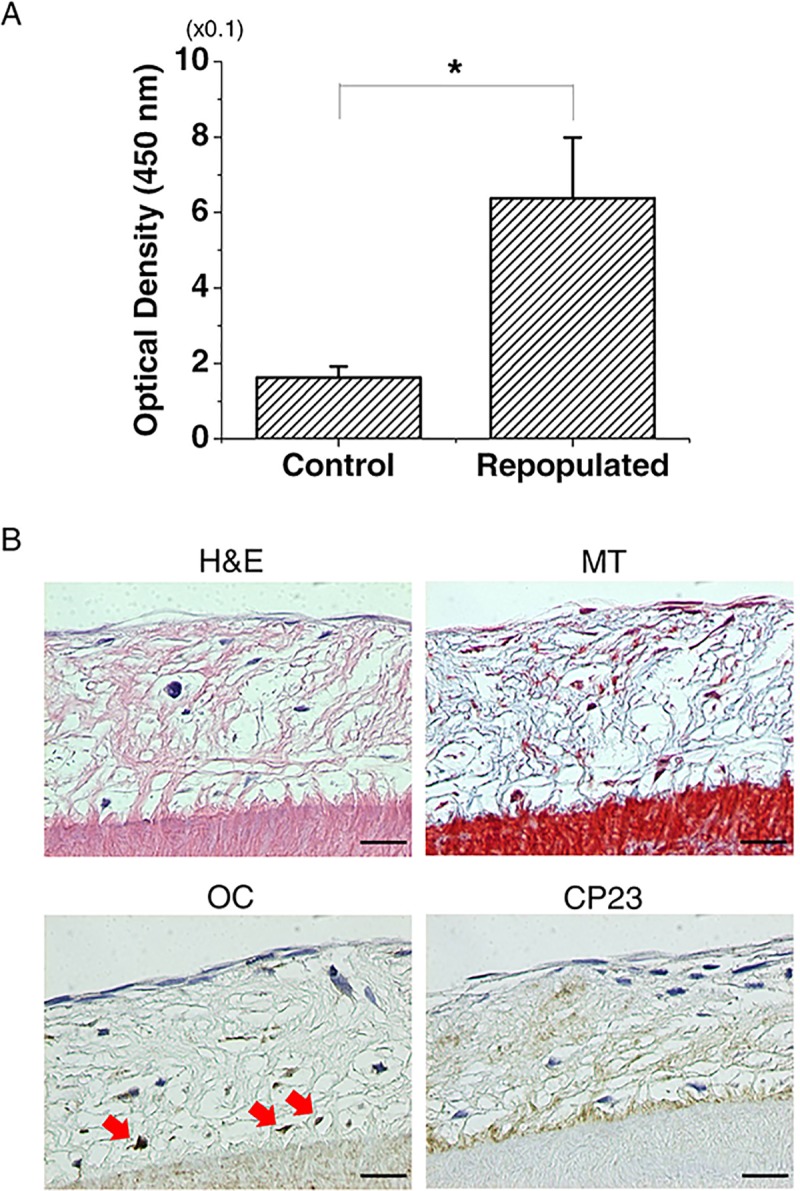
Characterization of decellularized human PDL periodontal ligament (dHPDL) recellularized with PDL stem cells (PDLSCs). (A) Cell viability for PII after recellularization. PDLSCs repopulated onto dHPDL when applying PII were viable compared with the control group. The Mann-Whitney U test (*p<0.05) was used to assess cell viability. (B) PDLSCs seeded onto decellularized tooth slices in PII repopulated and infiltrated toward the cementum (black arrows) in H&E and Masson’s trichrome (MT) staining. PDLSCs expressed osteocalcin (OC) and cementum protein 23 (CP23) near the cementum (red arrows) in recellularized human tooth slices in IHC staining. C, cementum. Scale bars: 20 μm.

PDLSCs were viable after 2 weeks of seeding PDLSCs onto decellularized scaffold for PII followed by culturing (Fig 3). PDL cells were repopulated on decellularized scaffold in H&E and Masson’s trichrome staining. Immunostaining of histological sections for OC showed positive staining in PDL cells adjacent to cementum, while significant CP23 staining was also found (Fig 3B).

### Differentiation of human PDLSCs in decellularized PDL

**Fig 4:**
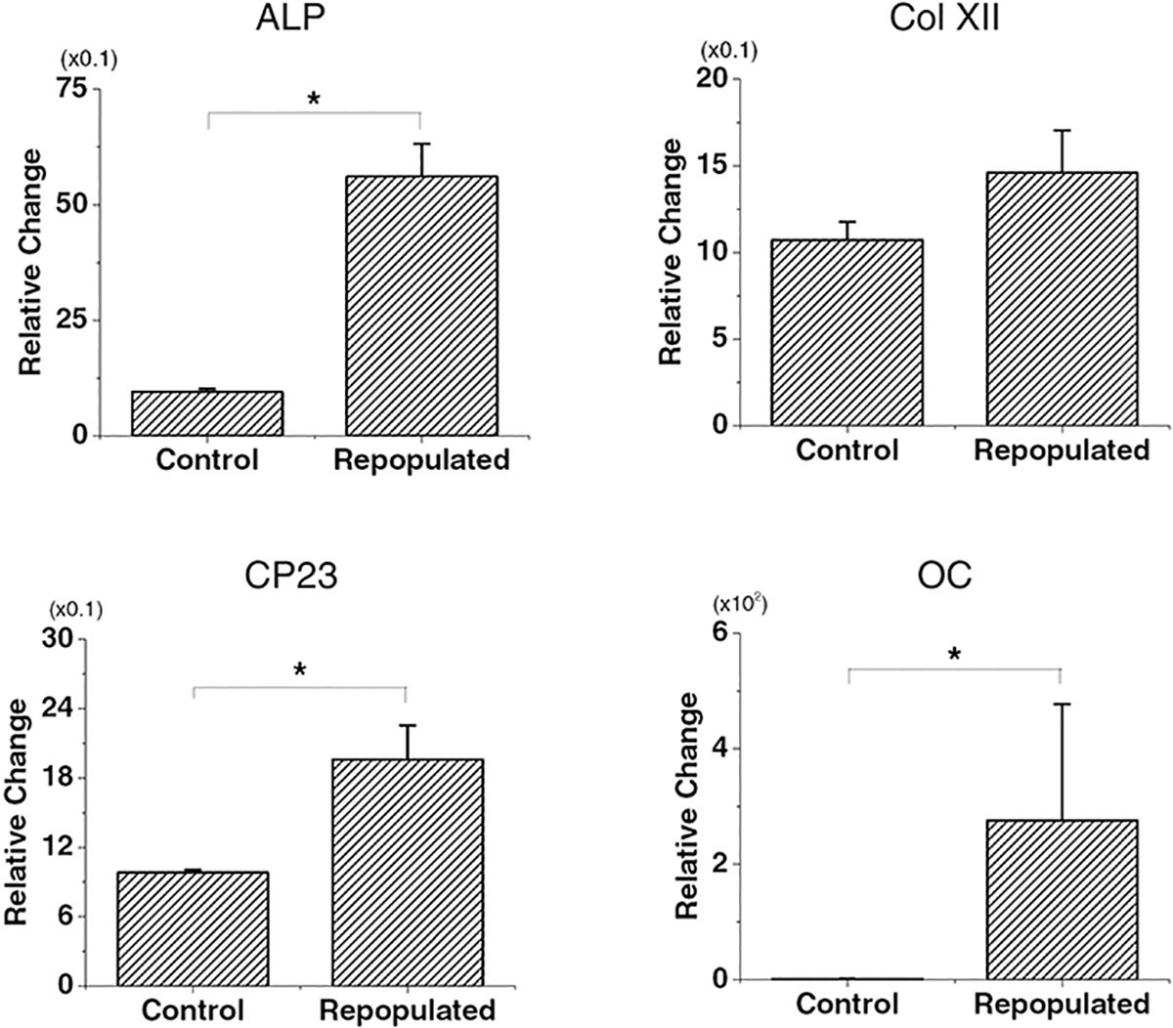
Gene expression in the recellularized human PDL group analyzed using the quantitative real-time reverse-transcription polymerase chain reaction. PDLSCs were seeded in the control group or in the decellularized group using PII. *p<0.05 for alkaline phosphatase (ALP) in Mann-Whitney U test; *p<0.05 for CP23 and OC in Student’s t-test.

Real-time PCR was performed to assess the gene expression of cementum/PDL markers (Table 2) by PDLSCs repopulated on decellularized PDL scaffolds of the tooth slices. The expression levels of alkaline phosphatase (ALP), CP23, and OC were significantly up-regulated after recellularization, by 5.61±0.70-, 1.96±0.30-, and 275.87±201.47-fold (mean±SD), respectively. Col XII was also up-regulated (by 1.46±0.24-fold), but the change was not statistically significant (Fig 4).

## Discussion

Decellularization protocols have been applied to various tissues and organs to fabricate bioactive ECM scaffold that could eliminate cells and cellular components completely without affecting the ECM [20–22]. Incompletely decellularized tissue might induce immune responses [23, 24], while a suitably tissue-engineered scaffold was found to not elicit immune responses in vivo [25]. The present study decellularized PDL in human tooth slices to assess the efficacy of nuclei and DNA removal while maintaining their native structure and composition. Furthermore, the recellularization potential of decellularized PDL scaffold was assessed by real-time PCR with the aid of periodontal tissue markers.

The successful application decellularization techniques might be dependent on many factors, including the types of tissue and organs, and the thickness, density, and cellularity of the tissue [26]. Previous studies found that using NH_4_OH and Triton X-100 could successfully remove cellular components [17, 26]. Decellularization with 1% Triton X-100 and 1% sodium dodecyl sulfate (SDS)**—**as used in PII in the present study—was effective in porcine anterior cruciate ligament and monkey kidney [27, 28]. Different methods using NH_4_OH, Triton X-100, hypertonic buffer, and SDS have recently been applied to human dental pulp tissue [16], but there had been no clinical investigation of decellularizing PDL obtained from human tooth slices. We applied a combination of NH_4_OH, Triton X-100, and SDS, which demonstrated effective decellularization compared to the other combinations of agents used in the previous studies.

In the present study, PII removed up to 62.32% of the DNA content, which was more than for PI. It seems that the SDS used in PII provided more complete removal of nuclear remnants than Triton X-100 [27, 28]. Ionic detergents such as SDS are more disruptive to tissue structure than is Triton X-100 [29]. However, the removal effectiveness was lower in the present study than in previous studies: at least 90% of host DNA was eliminated by SDS treatment in tissues and organs including rat forearm [30], porcine cornea [31], porcine heart valve [32], human vein [33], and human heart [34]. It appears that the specific characteristics of the tooth structure including its constituent hard tissue result in less elimination of hard tissue, and so additional agents for the complete removal of DNA (e.g., DNAase) need to be investigated in the future.

Sharpey’s fibers, which are the principal PDL fibers functioning in both tooth attachment and PDL regeneration, were shown to have a denser arrangement in PII. For evaluating the integrity of decellularized ECM, structural proteins such as collagen, fibronectin, and laminin as well as glycosaminoglycans should be evaluated [35]. Although fibronectin was notably decreased for PI and PII, Col I (which is the predominant type of collagen in PDL) was preserved in IHC staining, whereas Col XII was enhanced for PII. These findings could be attributed to teeth being involved in the mature/functional stage of PDL, as compared with developing stages, while the expression of Col I decreases with maturity [10, 36]. Furthermore, Col XII is responsible for the organization of collagenous fibers in response to mechanical loading in mature PDL [36]. The findings from both the present SEM and IHC analyses indicated that utilizing SDS and Triton X-100 preserved collagen fibrous network without any marked loss of structural proteins. This is consistent with [13] demonstrating the efficacy of 1% SDS and 1% Triton X-100 for the decellularization of heart-valve leaflets while preserving the integrity of the ECM scaffold.

Few studies have evaluated the influence of a decellularized scaffold on gene expression and cell differentiation. The gene expression markers selected in the present study are relevant to wound healing and the regeneration of bone, cementum, and PDL tissue (Table 2). The expression levels of CP23, ALP, and OC were higher than that of Col XII at 5 weeks after cell seeding. This could be explained by the induction of a later differentiation response of the repopulating PDL cells after the initial proliferative phase [18].

The human PDLSCs in this study were viable and able to be recellularized onto decellularized periodontal scaffold, as demonstrated by the cell viability analysis and H&E staining. OC expression was more prominent than CP23 expression in IHC staining. The expression of OC surrounding the cementum in the recellularized constructs suggests that decellularized scaffolds have the potential of inducing biomineralization and bone remodeling [7]. It is noteworthy that PDLSCs could be infiltrated and repopulated close to the cementum area from outside of the ECM.

Tooth avulsion accounts for 16% of all traumatic injuries in the permanent dentition, and it is accompanied by severe damage to the PDL [37]. PDL cells are damaged by inadequate storage of the avulsed teeth. Following the replantation of avulsed teeth, inflammatory root resorption and further tooth loss could occur. In this study, decellularized PDL scaffolds of tooth sections preserved their structure, and collagen remained in the ECM. They also have potential for recellularization and to induce the expression of genes related to PDL and bone regeneration. The application of autologous mesenchymal stem cells to the decellularized periodontal scaffold of avulsed tooth that potentially promotes the proliferation and differentiation of PDL cells could be a novel approach for PDL regeneration.
